# Enhanced breast cancer progression by mutant p53 is inhibited by the circular RNA circ-Ccnb1

**DOI:** 10.1038/s41418-018-0115-6

**Published:** 2018-05-23

**Authors:** Ling Fang, William W. Du, Juanjuan Lyu, Jun Dong, Chao Zhang, Weining Yang, Alina He, Yat Sze Sheila Kwok, Jian Ma, Nan Wu, Feiya Li, Faryal Mehwish Awan, Chengyan He, Bing L. Yang, Chun Peng, Helen J. MacKay, Albert J. Yee, Burton B. Yang

**Affiliations:** 1 Sunnybrook Research Institute, and Department of Laboratory Medicine and Pathobiology, University of Toronto, Toronto, Canada; 2 China-Japan Union Hospital of Jilin University, Jilin, China; 3 Sunnybrook Research Institute, Toronto, Canada; 4 Department of Biology, York University, Toronto, Canada

## Abstract

TP53 mutations occur in many different types of cancers that produce mutant p53 proteins. The mutant p53 proteins have lost wild-type p53 activity and gained new functions that contribute to malignant tumor progression. Different p53 mutations create distinct profiles in loss of wild-type p53 activity and gain of functions. Targeting the consequences generated by the great number of p53 mutations would be extremely complex. Therefore, in this study we used a workaround and took advantage of the fact that mutant p53 cannot bind H2AX. Using this, we developed a new approach to repress the acquisition of mutant p53 functions. We show here that the delivery of a circular RNA circ-Ccnb1 inhibited the function of three p53 mutations. By microarray analysis and real-time PCR, we detected decreased circ-Ccnb1 expression levels in patients bearing breast carcinoma. Ectopic delivery of circ-Ccnb1 inhibited tumor growth and extended mouse viability. Using proteomics, we found that circ-Ccnb1 precipitated p53 in p53 wild-type cells, but instead precipitated Bclaf1 in p53 mutant cells. Further experiments showed that H2AX serves as a bridge, linking the interaction of circ-Ccnb1 and wild-type p53, thus allowing Bclaf1 to bind Bcl2 resulting in cell survival. In the p53 mutant cells, circ-Ccnb1 formed a complex with H2AX and Bclaf1, resulting in the induction of cell death. We found that this occurred in three p53 mutations. These results shed light on the possible development of new approaches to inhibit the malignancy of p53 mutations.

## Introduction

Circular RNAs form covalently closed loops that can be produced from exons and introns [1–3]. Circularization of transcripts was long believed to be the result of erroneous splicing processes within cells. This idea has recently been challenged with the observation that circular RNAs can be detected extensively in an evolutionarily conserved manner [4–6]. Although circular RNAs are grouped as non-coding RNAs, some have been shown to code for protein peptides [7, 8]. Given their abundance and evolutionary conservation, it is likely that circular RNAs have potential regulatory roles [9–11]. In addition to their canonical structure and ability to bind proteins, circular RNAs may form complex three dimensional structures and conformations [1]. This allows circular RNAs to acquire additional impact on gene expression and protein binding, which is distinct from the mechanisms their analogous linear mRNA counterparts exert [12, 13]. In congruence with these facts, we recently reported that circ-Foxo3 represses tumor progression by binding to Mdm2 and p53 [14].

The tumor suppressor p53 is a transcription factor that contains 393 amino acids with two distinct nucleic acid-binding domains: the central DNA-binding core domain and a second nucleic acid-binding domain at the C-terminal (30 amino acids in size). The core domain is responsible for binding to DNA at target promoters, and it is a common locus where oncogenic missense mutations can occur. The C-terminal domain possesses RNA binding activity [15]. While most studies have reported hotspot mutations in p53, it in fact appears that mutations may occur in almost every codon within the DNA binding domain and other domains of p53, in cancer cells [16–20]. It is known that mutant p53 enhances cancer progression and malignancy [21, 22]. However, it is not known how different mutations affect cancer progression in loss of wild-type p53 function, dominant negative mutations, and gain of function phenotypes [23]. It is extremely complex to design approaches to target mutant p53 and/or the downstream signaling pathways. In this study, we describe a circular RNA circ-Ccnb1 that can bind to H2AX and wild-type p53, thus avoiding induction of cell death. However, in p53 mutant cells, circ-Ccnb1 forms a complex with H2AX and Bclaf1, resulting in cancer cell death and inhibition of tumor progression.

## Results

### Inhibitory effect of circ-Ccnb1 on breast cancer cell proliferation and survival

**Fig. 1:**
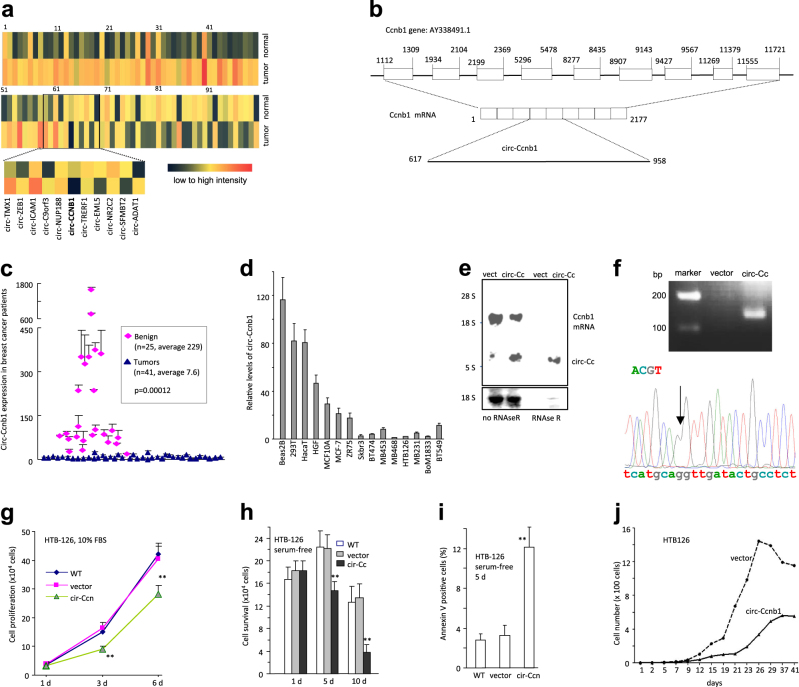
Circ-Ccnb1 was down regulated in breast cancer cells and inhibited cell proliferation and survival. **a** Comparison of circular RNA expression in human breast cancer relative to the adjacent benign tissues by using the Agilent Human circular RNA microarray. **b** Structures of Ccnb1 genome and transcript. Circ-Ccnb1 is produced by exons 4–5. **c** Real-time PCR showed that circ-Ccnb1 levels were significantly lower in the tumor tissues relative to the adjacent tissues. **d** Expression of circ-Ccnb1 in human non-cancer cell lines (Beas2B, 293T, HaCat, HGF, and MCF10A) and breast cancer cell lines (MCF-7, ZR75, Skbr3, BT474, MB453, MB468, HTB126, MB231, Bom1833, and BT549). **e** Total RNA extracted from mock- or circ-Ccnb1-transfected HTB126 cells were incubated with or without RNAse R at 37 °C for 10 min, followed by gel electrophoresis and Northern hybridization to confirm overexpression of circ-Ccnb1 and its resistance to RNAse R treatment. **f** Upper, RT-PCR showing product from vector- and circ-Ccnb1-transfected cells. Lower, the PCR product was purified, cloned and subject to sequencing. This confirmed the correct junction (arrow) sequence of circ-Ccnb1. **g** Wild-type, circ-Ccnb1- and vector-transfected HTB126 cells (1 × 10^4^) were inoculated in 12-well plates in DMEM containing 10% FBS. Cell numbers were counted on Days 1, 3, and 6. **p < 0.01. Error bars, SD (*n* = 4). **h** The cells (1.5 × 10^5^) were inoculated in 6-well plates in serum-free DMEM. Ectopic expression of circ-Ccnb1 decreased cell survival. **p < 0.01. Error bars, SD (*n* = 4). **i** The cells were cultured in serum-free medium for 5 days and subject to Annexin V staining and flow cytometry analysis. Expression of circ-Ccnb1 increased apoptosis. **p < 0.01. Error bars, SD (*n* = 4). **j** Cells were inoculated in 96-well plates to obtain one cell per well. Cell proliferation was monitored for up-to 41 days. Expression of circ-Ccnb1 decreased cell growth

By microarray, we analyzed the expression levels of different circular RNAs in breast carcinoma patients relative to the adjacent benign tissues (three pairs). Although most of the reported circular RNAs could be detected by microarray, only a small portion was differentially expressed with a 2-fold cut-off. We searched for those circRNAs from which the parental genes are known to function in cancer development. Amongst these differentially expressed circular RNAs, we found that circ-Ccnb1 was greatly down-regulated in cancer tissue (Fig. 1a, names of circRNAs provided in Fig S1a). The circular RNA circ-Ccnb1 is derived from exon 4 and exon 5 of the *CCNB1* gene (Fig. 1b). Ccnb1 is a regulator of cell mitosis. Higher levels of Ccnb1 are found in many cancers, especially breast cancer [24, 25]. We measured circ-Ccnb1 levels in 66 samples, including both cancer and benign, by real-time PCR using primers listed in Table S1. We detected significantly lower levels of circ-Ccnb1 in the cancer tissues relative to the benign samples (Fig. 1c). Conversely, the levels of Ccnb1 mRNA were higher in cancer samples relative to benign samples (Fig S1b). However, there was no direct correlation between circ-Ccnb1 and Ccnb1 mRNA in each pair of samples (Fig S1c). We further examined circ-Ccnb1 expression in a number of cell lines and found that the non-cancer cell lines expressed much higher levels of circ-Ccnb1 (Fig. 1d).

To explore whether circ-Ccnb1 could be developed as an agent for molecular therapy in cancer, we generated an expression construct expressing circ-Ccnb1 and a mock control (Fig S1d). Transfection of human breast cancer cell line HTB126 with circ-Ccnb1 increased circ-Ccnb1 levels, but had no effect on the expression of parental Ccnb1 mRNA (Fig S1e). To confirm circularization of the product, we treated RNAs extracted from circ-Ccnb1- and vector-transfected cells with or without RNase R. By Northern blotting, we confirmed that cells transfected with the construct expressed higher levels of circ-Ccnb1 than the vector control (Fig. 1e). By real-time PCR, we confirmed that while RNAse R treatment decreased Ccnb1 linear mRNA levels, it did not affect circ-Ccnb1 levels (Fig S1f). Further confirmation was done by RT-PCR, where we detected the expected size of PCR product (Fig. 1f, upper), and by DNA sequencing, which showed the exact junction sequence (Fig. 1f, lower).

We analyzed the phenotypic effects of circ-Ccnb1 expression and found that ectopic circ-Ccnb1 decreased proliferation (Fig. 1g) and survival (Fig. 1h), but increased apoptosis (Fig. 1i) of HTB126 cells. Using a single cell proliferation assay, we further examined the effect of circ-Ccnb1 on cell proliferation. Individual circ-Ccnb1- and vector-transfected cells were cultured in 96-well plates (1 cell/well). Quantification was performed for 41 days. Ectopic circ-Ccnb1 decreased the proliferative capacity of cancer cells relative to vector-transfected cells (Fig. 1j). We designed two siRNAs specifically targeting circ-Ccnb1, but had no effect on linear Ccnb1 mRNA (Fig S2a), to test the role of endogenous circ-Ccnb1 in cancer cells (MB231 cells). We confirmed that silencing circ-Ccnb1 increased proliferation (Fig S2b), survival (Fig S2c), but decreased apoptosis (Fig S2d). While silencing circ-Ccnb1 increased MB231 proliferation, silencing Ccnb1 mRNA decreased proliferation (Fig S2e). The effect of circ-Ccnb1 on apoptosis appeared to be the major cause of cell survival, since addition of Caspase-1 inhibitor abolished the effect of circ-Ccnb1 on cell cycle progression (Fig S2f).

Silencing circ-Ccnb1 was also performed in a non-cancer cell line 293T. After confirming the silencing of circ-Ccnb1 (Fig S2g), we unexpectedly observed decreased cell proliferation (Fig S2h) and survival, but increased apoptosis in the 293T cells (Fig S2i). In 293T cells, silencing circ-Ccnb1 and Ccnb1 mRNA decreased cell proliferation (Fig S2j).

### Circ-Ccnb1 interacted with H2AX, p53, and Bclaf1

**Fig. 2:**
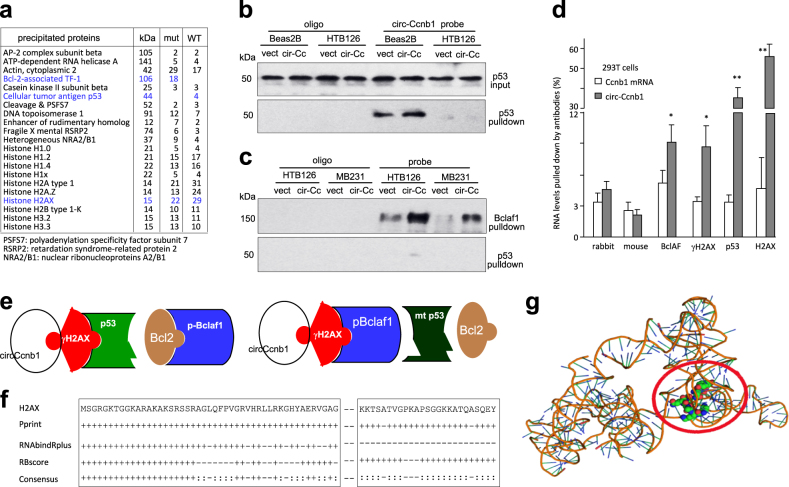
circ-Ccnb1 interacts with H2AX. **a** The circ-Ccnb1 probe pulled-down proteins were subject to proteomic assay. In the Beas2B cells, H2AX and p53 were precipitated. In the HTB126 cells, H2AX and pBclaf1 were precipitated. The last two columns represent the number of pulled down peptides detected by the system. **b** Lysates prepared from vector- or circ-Ccnb1-transfected HTB126 and Beas2B cells were subject to probe pull-down followed by Western blotting. The circ-Ccnb1 probe precipitated p53 in Beas2B cells. **c** In lysates prepared from vector- or circ-Ccnb1-transfected HTB126 and MB231 cells, the probe could not precipitate p53, but could precipitate Bclaf1. Ectopic expression of circ-Ccnb1 increased Bclaf1 precipitation. **d** Lysate prepared from 293T cells was incubated with antibodies as shown, followed by real-time PCR for levels of linear Ccnb1 mRNA and circ-Ccnb1. Anti-H2AX, p53, γH2AX, and Bclaf1 antibodies pulled-down circ-Ccnb1, but not linear Ccnb1 mRNA. **p < 0.01. Error bars, SD (*n* = 4). **e** Models depicting circ-Ccnb1 interacting with H2AX, p53 and Bclaf1. **f** Prediction of probable RNA-binding residues of H2AX was carried out by submitting the H2AX sequence to Pprint, RNABindRPlus and RBscore servers. “+” indicates the predicted RNA-binding residues. **g** Graphical representation of three-dimensional structures of the docking models of circ-Ccnb1 with the binding fragment of H2AX by NPDock

Our previous report demonstrated that circ-Foxo3 is highly expressed in the cytosol and can regulate miRNA functions [26]. We analyzed circ-Ccnb1 and found that it is expressed at low levels (Fig S3a), and a large portion is translocated to the nuclei (Fig S3b). Thus, it is unlikely for circ-Ccnb1 to function as a sponge for miRNA binding. To examine how circ-Ccnb1 functioned, we performed a protein precipitation assay using a probe specifically binding to circ-Ccnb1. The precipitated materials were subject to proteomic analysis. Among the many proteins revealed, we found that p53 was only precipitated in Beas2B cells that express wild-type p53, whereas Bclaf1 was only precipitated in HTB126 cells that express mutant p53 (Fig. 2a). Bclaf1 has shown to play roles in cancer development [27]. Our results suggested that circ-Ccnb1 does not directly bind to Bclaf1. We also detected precipitation of H2AX in both samples, since H2AX was reported to bind Bclaf1 and wild-type p53 [28]. H2AX functions in DNA repair and cell mitosis [29]. We validated this result in HTB126 cells and Beas2B cells and found that the circ-Ccnb1 probe only pulled down p53 in Beas2B cells (Fig. 2b). In the p53 mutant cells, HTB126 and MB231 cells, overexpression of circ-Ccnb1 allowed circ-Ccnb1 probe to pull down more Bclaf1, but not mutant p53 (Fig. 2c). We then performed a binding assay using antibodies against H2AX, γH2AX, p53, and Bclaf1 (Fig. 2d) to precipitate circ-Ccnb1 in 293T cells (wild-type p53). We detected precipitation of circ-Ccnb1, but not the Ccnb1 mRNA.

Based on these results, we proposed a model of circ-Ccnb1-protein interaction (Fig. 2e). Since Bclaf1 is a H2AX-dependent tumor suppressor [28], high levels of circ-Ccnb1 would bind H2AX and wild-type p53, blocking the tumor suppressing effect of p53 and allowing Bcl2 to bind Bclaf1. As a result, cells grew. However, mutant p53 cannot bind H2AX, which allowed Bclaf1 to bind H2AX. Binding of Bclaf1 to H2AX activated the tumor suppressing effects of Bclaf1, thus inducing cell death.

We analyzed the potential interaction of circ-Ccnb1 with H2AX using a computer algorithm. It appeared that the RNA-binding residues were concentrated in the N-terminal region and in the C-terminal domain of H2AX (Fig. 2f). The predicted secondary structure of circ-Ccnb1 was obtained by analyzing thermodynamic properties using the formula Δ*G* = Δ*H*–*T*Δ*S*, Δ*G* = −69.60 kcal/mol at 37 °C, Δ*H* = −886.90 kcal/mol, and Δ*S* = −2635.1 cal/(K mol), where *T*(K) is the absolute temperature and Δ*G*, Δ*H*, Δ*S*, denote the change in free energy, enthalpy, and entropy, respectively. The secondary structure delineated in dot bracket notation was then analyzed by the software RNA composer for tertiary structure prediction. Finally, NPDock was used to carry out the in silico molecular docking of circ-Ccnb1 with H2AX (Fig. 2g). The structure of γH2AX C-terminal peptide used in the docking procedure was derived from Protein Data Bank (PDB) entry 3SQD. The molecular simulation results predicted a minimal binding region of circ-Ccnb1 for H2AX as “uca” “ga” “ca” “aa” “uga” “u” (Fig S3c). The contact map (Fig S3d), the residue-level resolution contact maps (Fig S3e), the MC score (Fig S3f), the contact distance (Tables S2–3), the Accessible Surface Area (Table S4), and interaction overview (Table S5) all supported the conclusion that circ-Ccnb1 could dock the H2AX C-terminal regulatory domain.

### Effects of H2AX on mediating circ-Ccnb1 functions

**Fig. 3:**
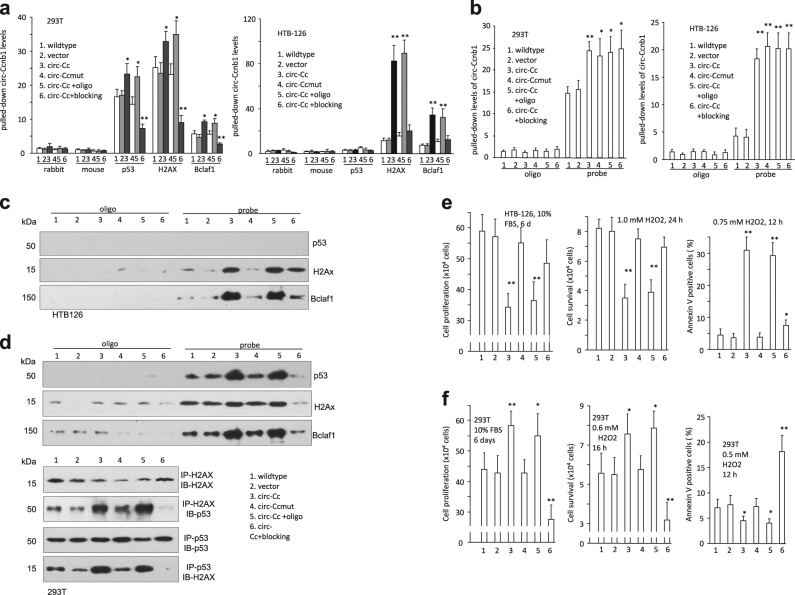
Effects of circ-Ccnb1 interacting with H2AX. **a** In 293T cells (left), anti-p53, H2AX, and Bclaf1 antibodies precipitated higher levels of circ-Ccnb1 in the circ-Ccnb1-transfected cells relative to the controls, which was abolished by transfection with the mutant circ-Ccnb1. Transfection with the blocking oligo inhibited this activity. In HTB126 cells (right), antibodies against H2AX and Bclaf1, but not p53, precipitated higher levels of circ-Ccnb1 in the circ-Ccnb1-transfected cells, which was abolished by transfection with the circ-Ccnb1 mutant and the blocking oligo (*n* = 4). **b** In 293T (left) and HTB126 (right) cells, transfection with the circ-Ccnb1 or the blocking oligo did not affect precipitation by the circ-Ccnb1 probe. **c** Pulling down circ-Ccnb1 from HTB126 lysate also precipitated H2AX and Bclaf1, which was abolished by transfection with circ-Ccnb1 mutant or the blocking oligo. Pulling down circ-Ccnb1 did not pull down p53. **d** Upper, pulling down circ-Ccnb1 from 293T lysate precipitated H2AX, Bclaf1 and p53, which was abolished by transfection with circ-Ccnb1 mutant or the blocking oligo. Lower, H2AX precipitation pulled down p53, and p53 precipitation pulled down H2AX, which was abolished by transfection with circ-Ccnb1 mutant or the blocking oligo. **e** Expression of circ-Ccnb1 repressed HTB126 cell proliferation (left) and survival (middle), and enhanced H2O2-induced apoptosis (right), which could be abolished by transfection with the mutant circ-Ccnb1 or the blocking oligo. **f** Expression of circ-Ccnb1 enhanced 293T cell proliferation (left) and survival (middle), and repressed apoptosis (right), which could be abolished by transfection with the mutant circ-Ccnb1 or the blocking oligo. [switch e and f legends]

To evaluate the effects of the binding sites on mediating circ-Ccnb1 functions, we generated circ-Ccnb1 mutations abolishing the interaction with H2AX by site-directed mutagenesis and designed blocking oligos complementary to the binding sites (Fig S3c). In 293T, but not HTB126 cells, anti-p53 antibody could precipitate circ-Ccnb1, which was abolished when the H2AX binding site was mutated or in the presence of the blocking oligo (Fig. 3a, sequences of control oligo and blocking oligo provided in Table S1). Nevertheless, the antibody against H2AX or Bclaf1 could precipitate circ-Ccnb1 in both wild-type and mutant p53-containing cells.

In the probe pull down assay, we observed that transfection with the circ-Ccnb1 mutant construct or the blocking oligo did not affect circ-Ccnb1 expression (Fig S4a) or the interaction between the probe and circ-Ccnb1 in both 293T and HTB126 cells (Fig. 3b). In HTB126 cells, pulling down circ-Ccnb1 precipitated H2AX and Bclaf1, but not mutant p53, while protein levels were not affected (Fig S4b); however, the interaction was abolished by transfection with the mutant circ-Ccnb1 or the blocking oligo (Fig. 3c). In 293T cells, pull down of circ-Ccnb1 precipitated H2AX, Bclaf1, and p53, which could be abolished by transfection with the mutant circ-Ccnb1 or the blocking oligo (Fig. 3d). Transfection with the mutant construct decreased precipitation of p53 by H2AX antibody, and vice versa. Transfection with the blocking oligo abolished precipitation of p53 by H2AX antibody, and vice versa. These results suggest that the interaction of circ-Ccnb1 was essential for H2AX to interact with wild-type p53.

In functional tests, we found that transfection with the mutant circ-Ccnb1 abolished the repressing effect of circ-Ccnb1 on HTB126 cell proliferation and survival, and enhanced apoptosis (Fig. 3e), causing increased cell proliferation and survival, and decreased apoptosis. Delivering a blocking oligo partially abolished the functions of circ-Ccnb1 in HTB126 cells. In 293T cells, transfection with the blocking oligo inhibited cell proliferation and survival, but increased apoptosis (Fig. 3f).

We also examined the roles of circ-Ccnb1 in cell migration and colony formation in two other cancer cell lines that contain wild-type p53 (MCF-7) and mutant p53 (MDA-MB-231). We detected that ectopic circ-Ccnb1 increased cell migration (Fig S4c) and colony formation (Fig S4d) in MCF-7 cells, but decreased cell migration (Fig S4e) and colony formation (Fig S4f) in MB231 cells. Silencing endogenous circ-Ccnb1 decreased cell migration (Fig S4g) in MCF-7 cells, but increased cell migration (Fig S4h) in MB231 cells.

**Fig. 4:**
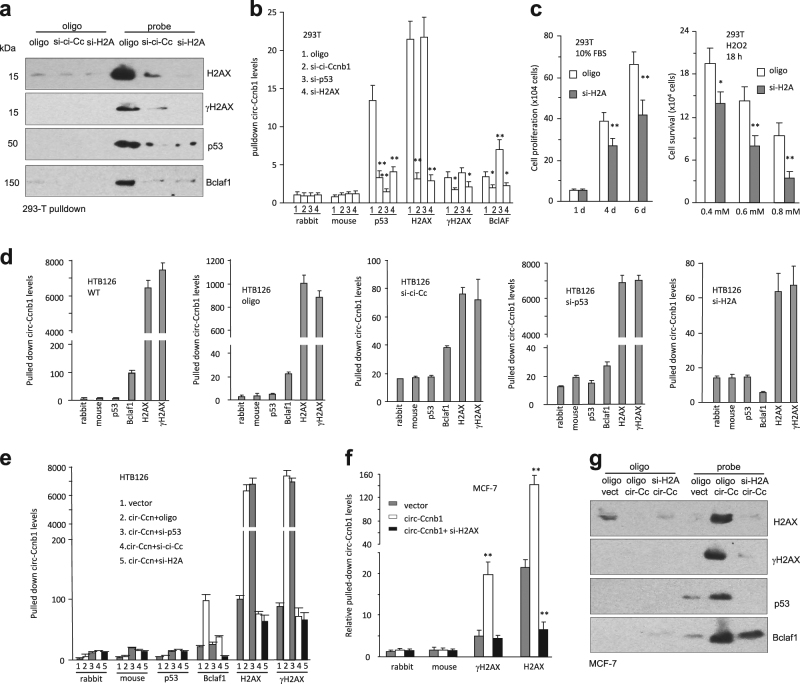
Silencing H2AX abolished circ-Ccnb1 function in p53 mutant cells. **a** Western blot showed that circ-Ccnb1 probe could precipitate H2AX, γ-H2AX, p53, and Bclaf1, but could not pull down these proteins after silencing circ-Ccnb1 or H2AX. **b** Antibodies against p53, H2AX, γ-H2AX, or Bclaf1 pulled down circ-Ccnb1 from 293T lysate, which was prevented by silencing circ-Ccnb1 and H2AX. **c** Silencing H2AX repressed 293T cell proliferation in 10% FBS/DMEM medium (left) and survival after treatment with H2O2 for 18 h (right). **d** HTB126 lysate or lysate from cells transfected with the control oligo, circ-Ccnb1 siRNA, p53 siRNA, and H2Ax siRNA, were subjected to pull down with antibodies against p53, Bclaf1 H2AX, and γ-H2AX. Silencing H2AX decreased pulling down circ-Ccnb1 by Bclaf1 antibody, but silencing p53 did not. **e** Silencing p53 increased anti-H2AX antibody pulling down circ-Ccnb1. **f** In cross-linking immunoprecipitation assay, antibodies against H2AX and γH2AX could precipitate circ-Ccnb1. Silencing H2AX decreased precipitation. **g** Western blot showed that circ-Ccnb1 probe pulled down p53, H2AX, γ-H2AX, and Bclaf1. Silencing H2AX abolished pulling down of p53, H2AX, and γ-H2AX, but had little effect on Bclaf1 precipitation

We then silenced H2AX in 293T cells using the siRNA approach and observed that silencing H2AX did not affect expression of the other proteins (Fig S5a), but abolished precipitation of p53 and Bclaf1 by circ-Ccnb1 (Fig. 4a). Nevertheless, silencing H2AX did not affect circ-Ccnb1 expression or circ-Ccnb1 precipitation by the circ-Ccnb1 probe (Fig S5b). Silencing H2AX abolished p53 pulling down circ-Ccnb1, but silencing p53 did not affect H2AX pulling down circ-Ccnb1 (Fig. 4b). Interestingly, silencing p53 caused increased circ-Ccnb1 to be pulled down by Bclaf1. This suggests that p53 was competing with Bclaf1 for binding circ-Ccnb1. The former appeared to have higher affinity for circ-Ccnb1. Silencing H2AX inhibited cell proliferation and survival (Fig. 4c). This phenomenon was also observed in two other p53 wild-type cell lines BEAS2B and MCF-10A (Fig S5c).

To further validate our proposed model, we performed the experiments in p53 mutant cell line HTB126. We observed that silencing mutant p53 had no effect on circ-Ccnb1 precipitation by the other proteins, suggesting that the mutant p53 was not involved in circ-Ccnb1 binding to the other proteins (Fig. 4d). However, silencing H2AX decreased Bclaf1 pulling down circ-Ccnb1, suggesting the formation of a circ-Ccnb1–H2AX–Bclaf1 complex. In the circ-Ccnb1-transfected cells, silencing H2AX abolished Bclaf1 pulling down circ-Ccnb1, while silencing p53 had no effect on H2AX pulling down circ-Ccnb1 (Fig. 4e).

In the p53 wild-type tumor cell line MCF-7, silencing H2AX did not affect the circ-Ccnb1 probe pulling down circ-Ccnb1 (Fig S5d). It also did not affect expression of Bclaf1 and p53 (Fig S5e). In cross-linking immunoprecipitation assay, high levels of circ-Ccnb1 were precipitated by antibodies against H2AX and γH2AX (Fig. 4f). The circ-Ccnb1 probe precipitated H2AX, γH2AX, p53, and Bclaf1. Silencing H2AX abolished circ-Ccnb1 probe pulling down H2AX, γH2AX, and p53, but had no effect on circ-Ccnb1 probe pulling down Bclaf1 (Fig. 4g).

### Distinction of wild-type and mutant p53 on mediating circ-Ccnb1 effects

**Fig. 5:**
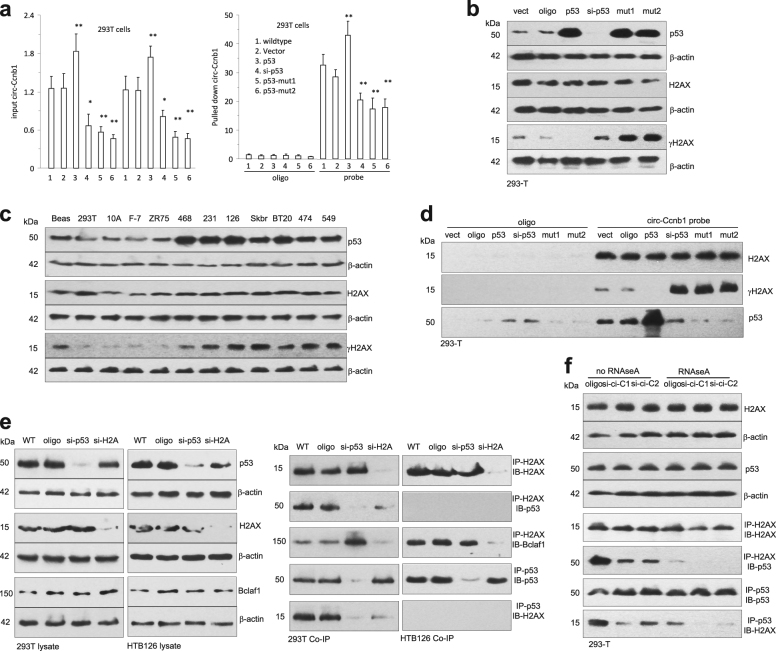
Effect of p53 on the interaction of circ-Ccnb1 with H2AX and Bclaf1. **a** Real-time PCR showed circ-Ccnb1 levels in the input (left) and levels pulled down by the probe from 293T cells (right). Expression of p53 enhanced circ-Ccnb1 expression, while silencing or the expression of mutated p53 repressed circ-Ccnb1 levels. **b** In Western blotting, ectopic wild-type p53 repressed γH2AX levels, while silencing p53 or transfection with mutant p53 enhanced γH2AX expression. **c** Western blotting showed that the mutant p53 cell lines (MB-468, MB231, HTB126, SK-BR-3, BT-20, BT-474, and BT-549) expressed high levels of γ-H2AX. **d** Pull-down assay showed that precipitation of circ-Ccnb1 pulled down more γH2AX and less p53 in the cells when p53 was silenced or transfected with a p53 mutant construct. **e** Cell lysates from 293T (left) and HTB126 (right) were subject to Western blotting and immunoprecipitation with anti-H2AX and anti-p53 antibodies. Anti-H2AX antibody could precipitate p53 and Bclaf1. Precipitating p53 also pulled down H2AX in the 293T cell line, but not in the HTB126 cells. **f** Lysates from 293T cells were subject to Western blotting and immunoprecipitation with antibodies against H2AX and p53. H2AX precipitation pulled down p53, and precipitating p53 also pulled down H2AX, which could be prevented by silencing circ-Ccnb1 or treatment with RNAse A

To validate the effect of p53 mutation on the binding of circ-Ccnb1 to other proteins and functions, we employed constructs expressing wild-type p53 (plenti6/V5-p53_wt from Addgene) and mutant p53 (plenti6/V5-p53_R280K and plenti6/V5-p53_R175H). We observed that ectopic wild-type p53 enhanced circ-Ccnb1 expression, whereas silencing wild-type p53 or the expression of mutated p53 repressed circ-Ccnb1 expression levels (Fig. 5a). This result suggests that the interaction of wild-type p53-H2AX-circ-Ccnb1 might form a complex that increased the stability of circ-Ccnb1. We also observed that ectopically expressed wild-type p53 decreased γH2AX levels, while silencing p53 or transfection with mutant p53 enhanced γH2AX expression (Fig. 5b). To support this result, we analyzed γH2AX levels in p53 wild-type and mutant cell lines. We observed that most of the p53 wild-type cell lines expressed lower levels of γH2AX than the p53 mutant cell lines (Fig. 5c). In pull down assays, silencing p53 or transfection with mutant p53 facilitated circ-Ccnb1 pulling down γH2AX, which was abolished by transfection with the wild-type p53 (Fig. 5d).

We tested the formation of the circ-Ccnb1–H2AX–Bclaf1 complex. In 293T cells, silencing p53 abolished H2AX pulling down p53 and vice versa (Fig. 5e). However, silencing p53 increased H2AX pulling down Bclaf1. In HTB126 cells, since mutant p53 does not bind H2AX, with or without silencing p53 or H2AX, they did not interact with each other. We also used RNAse-A to cleave circ-Ccnb1. Silencing endogenous circ-Ccnb1 decreased H2AX pulling down p53 and vice versa (Fig. 5f). Cleavage of circ-Ccnb1 displayed the same effects.

**Fig. 6:**
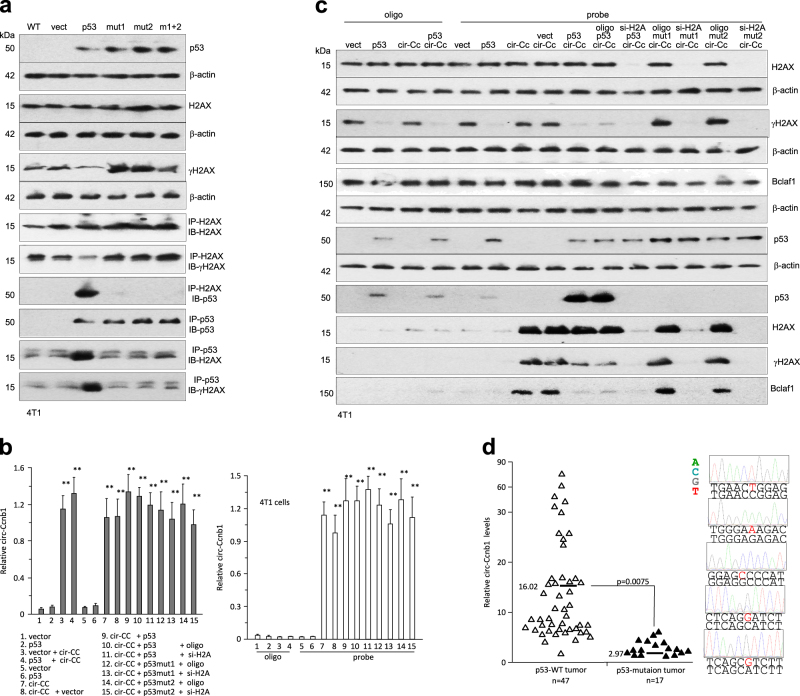
Expression of p53 repressed γ-H2AX. **a** Lysates prepared from 4T1 cells were subject to Western blotting and immunoprecipitation with antibodies against H2AX and p53. H2AX precipitation pulled down p53, and p53 precipitation pulled down H2AX, only when p53 is wild-type. **b** Lysates from 4T1 cells were subject to circ-Ccnb1 pull-down assay. Real-time PCR showed the levels of circ-Ccnb1 in the input (left) and precipitation mixture (right). **c** Pull-down assay showed that precipitation of circ-Ccnb1 pulled down H2AX and γH2AX in the presence of wild-type p53, but it precipitated H2AX, γH2AX, and Bclaf1 in the presence of mutant p53. **d** Left, circ-Ccnb1 levels were lower in the p53 mutation tumors compared to tumors of wild-type p53. Right, five typical p53 mutation sequences are shown

We further tested the roles of wild-type and mutant p53 in the cell line 4T1, which does not express p53. Transfection of wild-type p53 decreased γH2AX levels, while transfection with the mutant constructs promoted γH2AX expression (Fig. 6a). Transfection with wild-type p53 allowed H2AX to pull down p53 and vice versa. These interactions did not occur when the cells were transfected with the mutant p53. We confirmed that transfection with the wild-type or mutant p53 did not affect circ-Ccnb1 expression (Fig. 6b, left), or its interaction with the circ-Ccnb1 probe (Fig. 6b, right). In the pull-down assay, we showed that precipitation of circ-Ccnb1 pulled down H2AX, γH2AX, and p53 when the cells were transfected with wild-type p53, but it pulled down H2AX, γH2AX, and Bclaf1 when the cells were transfected with mutant p53 (Fig. 6c). The presence of p53 competed with Bclaf1 for binding to H2AX.

We examined the relevance of p53 mutation and circ-Ccnb1 expression in breast carcinoma patients. RNAs isolated from the tumor samples were analyzed for circ-Ccnb1 levels and sequencing for mutation of p53. We detected p53 mutation in 17 patients from a total of 64 patient samples. Analysis of circ-Ccnb1 levels showed that the p53 mutation samples displayed significantly lower levels of circ-Ccnb1, compared to the p53 wild-type samples (Fig. 6d).

### Circ-Ccnb1 decreased tumor xenograft growth and increased mouse survival

**Fig. 7:**
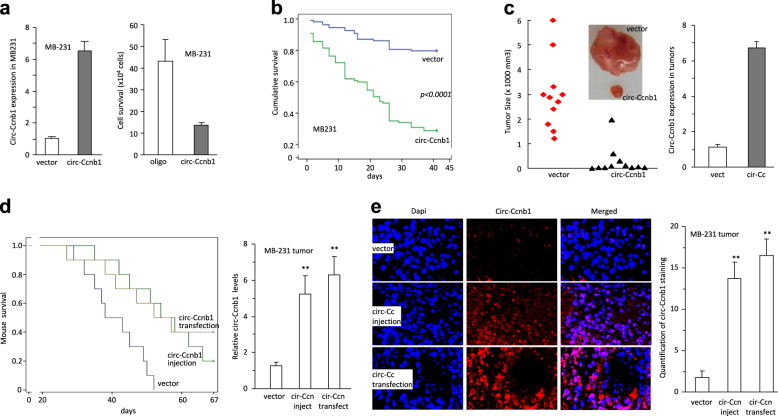
Expression of circ-Ccnb1 repressed tumor progression in p53 mutant cells. **a** Left, Transfection of circ-Ccnb1 enhanced circ-Ccnb1 expression in MB231 cell line. Right, expression of circ-Ccnb1 repressed cell survival in serum-free medium. **b** Cells were inoculated in 96-well plates to obtain one cell per well. Cell survival was monitored for up-to 45 days. Expression of circ-Ccnb1 decreased cell viability. **c** Circ-Ccnb1- and vector-transfected MB231 cells were subcutaneously injected into nude mice. Expression of circ-Ccnb1 repressed tumor growth (left). Real-time PCR showed that tumors from circ-Ccnb1-transfected cells expressed higher levels of circ-Ccnb1 relative to control. **d** Left, Nude mice were intraperitoneally injected with MB231 cells transfected with a control vector or the circ-Ccnb1 plasmids, or injected with MB231 cells and the circ-Ccnb1 plasmids. Compared to mice injected with the control vector, injection with circ-Ccnb1 plasmids or with the circ-Ccnb1-transfected MB231 cells, increased mouse survival significantly. Right, Real-time PCR showed increased circ-Ccnb1 levels in the tumors injected with the circ-Ccnb1 plasmids or formed by the circ-Ccnb1-transfected cells relative to the control. **e** Left, In situ hybridization showed that significantly higher levels of circ-Ccnb1 were detected in the tumors of mice injected with circ-Ccnb1 plasmids or circ-Ccnb1-transfected cells, relative to the control. Right, quantification of hybridization staining showed increased circ-Ccnb1 levels in the tumors injected with the circ-Ccnb1 plasmids or formed by the circ-Ccnb1-transfected cells relative to the control

We tested the inhibitory effect of circ-Ccnb1 on breast cancer cell growth using a different p53 mutant cell line MDA-MB-231. Ectopic transfection of the circ-Ccnb1 construct promoted circ-Ccnb1 levels, resulting in decreased cell survival (Fig. 7a). Individual cells were cultured in a density of one cell per well. After 45 days of culturing, the cumulative survival decreased significantly when the cells were stably transfected with circ-Ccnb1 relative to the control vector (Fig. 7b). The cells were subject to tumor xenograft in nude mice. Ectopic expression of circ-Ccnb1 repressed tumor growth significantly (Fig. 7c, left). Real-time PCR showed that the tumor tissues from circ-Ccnb1-transfected cells expressed higher levels of circ-Ccnb1 relative to control (Fig. 7c, right).

Nude mice were injected intraperitoneally with MDA-MB-231 cells transfected with or without the circ-Ccnb1 construct. The mice injected with the un-transfected MDA-MB-231 cells were injected with circ-Ccnb1 expression plasmids, followed by survival test. Kaplan-Meier survival curves showed that the mice injected with the circ-Ccnb1-transfected cells and the mice injected with MDA-MB-231 cells followed by injection of the circ-Ccnb1 expression plasmids, survived longer than the mice injected with vector-transfected MDA-MB-231 cells **(**Fig. 7d, left). Analysis of tumor masses showed that significant up-regulation of circ-Ccnb1 was detected not only in the tumors formed by circ-Ccnb1-transfected cells, but also in the tumors where circ-Ccnb1 expression plasmids were injected (Fig. 7d, right). This suggested intake of circ-Ccnb1 expression plasmids into the tumor cells. To examine this directly, we analyzed tumor sections by in situ hybridization and confirmed the presence of high levels of circ-Ccnb1 in both groups of tumors, reaching a *p*-value of significance (Fig. 7e).

## Discussion

To explore the involvement of circular RNAs in breast cancer development, we analyzed levels of circular RNAs in human breast cancers and benign tissues by microarray. We found that a particular circular RNA circ-Ccnb1 was down-regulated in cancer samples. However, due to the limited number of samples, it is not clear whether there is an association between circ-Ccnb1 levels and patient survival. We then found that circ-Ccnb1 could bind H2AX and wild-type p53, facilitating p53 wild-type cell proliferation and survival. Importantly, circ-Ccnb1 formed a complex with H2AX and Bclaf1, but not mutant p53, which induced p53 mutant cell death. Mutations in p53 have been found to produce distinct profiles in terms of loss of wild-type p53 function and gain of mutant p53 function [30, 31]. Since p53 mutations can occur in every codon of the DNA binding domain and even outside this domain, there are varieties of malignant consequences that can result from the many possible p53 mutations. It is highly complicated to design therapeutic approaches to target the mutations or the downstream signaling pathway of p53. However, we took a different approach that did not target mutant p53 or the downstream effectors, but instead took advantage of the fact that mutant p53 can no longer bind Bclaf1. It has been known that Bclaf1 is a H2AX-dependent tumor suppressor [28]. We found that in the p53 mutant cells, circ-Ccnb1 formed a complex with H2AX and Bclaf1, facilitating cancer cell death. This only occurs in the p53 mutant cells, because in the wild-type p53 cells, wild-type p53 has a greater affinity to bind H2AX, thus leaving Bclaf1 to bind Bcl2. The binding of Bclaf1 with Bcl2 will facilitate cell proliferation [32]. This binding is disrupted in the presence of mutant p53, where Bclaf1 can interact with H2AX and circ-Ccnb1 to induce death of p53 mutant cancer cells.

Thus, circ-Ccnb1 has no effect on p53 wild-type cells, as it in fact works to enhance p53 wild-type cell proliferation and survival. This is important because this potential agent will have minimal effects on the surrounding stromal tissues and healthy cells of patients who are undergoing cancer therapy using this approach. The specific targeting of mutant p53 cancer cells makes this circular RNA a great candidate for the development of new potential therapeutic approaches.

It has been known that the p53 mutant cells express much higher levels of the mutant p53 [33]. We confirmed this result by comparing the levels of mutant p53 in seven p53 mutant breast cancer cell lines with five p53 wild-type cell lines. It was thought that these very high levels of mutant p53 make these mutant proteins very attractive as therapeutic targets. However, for these very high levels of mutant proteins, large amounts of targeting molecules will be needed to sufficiently block the functions of the mutant proteins. In our studies, we selected to leave the mutant proteins alone. The fact that mutant p53 can no longer bind H2AX, which allows Bclaf1 to bind H2AX in the presence of circ-Ccnb1, provides a great advantage to induce the death of the p53 mutant cells. In this way, the therapy will not depend on how many targeting molecules are used or how much mutant p53  is present in a cell. As long as circ-Ccnb1 can form a complex with H2AX and Bclaf1, the p53 mutant cells will undergo apoptosis.

In this study, we used HTB126 breast cancer cells as a model to examine the effects of circ-Ccnb1 on p53 mutant cells. The p53 mutation in the HTB126 cell line occurs by substitution of valine to phenylalanine at codon 157 in exon 5. This missense mutation substitutes a smaller hydrophobic amino acid with a larger hydrophobic amino acid phenylalanine. This mutation alters the packing of the hydrophobic interface between the beta-sheets [34]. Specifically, it has been shown to affect the β-sheets and local packing in the central DNA core domain [35]. Furthermore, we have also employed two additional p53 mutant constructs to test the effects of circ-Ccnb1 on binding H2AX and Bclaf1, and examined its role in cancer cell death. While results obtained from these three p53 mutant cells were consistent, we do not know whether circ-Ccnb1 expression construct can also inhibit tumor progression of other p53 mutant cells in a similar way to the three mutations examined in this study. It would be interesting to test the role of circ-Ccnb1 in other p53 mutant cancer cells.

To explore the possibility of using circ-Ccnb1 as an agent for cancer therapy, we developed a peritoneal cancer model by injecting cancer cells into the peritoneal cavity of mice. This way, the tumor cells can readily spread everywhere in the cavity forming a large number of small tumors. This approach mimics a number of cancers such as serous ovarian carcinoma and lung cancer. It allows easy uptake of nanoparticle conjugated circ-Ccnb1 expression plasmids. For large solid tumors, the efficiencies of plasmid uptake are significantly lower than these small tumors. Advanced techniques need to be developed to allow effective delivery of plasmids, such as circ-Ccnb1, into solid tumors for future cancer therapy.

## Methods

### Microarray of samples from breast cancer patients

The RNA isolation and microarray analysis of human circular RNAs was performed by KangChen BioTech (Shanghai). Total RNAs were isolated from three pairs of pooled samples and digested with RNAse R (Epicentre, Inc.) to remove linear RNAs. The enriched circular RNAs were amplified and transcribed into fluorescent cDNA utilizing a random priming method and hybridized onto the Arraystar Human circRNA Array V2. Three samples were grouped for array analysis. Circular RNAs were selected based on being significantly differentially expressed (fold changes ≥2 and p values ≤ 0.05). Consent for human samples was obtained according to the Declaration of Helsinki.

### Construct generation

A construct expressing human circular RNA Ccnb1 (circ-Ccnb1) was generated by us. The plasmids contained a Bluescript backbone, a CMV promoter driving mouse circ-Ccnb1 or a non-related sequence serving as a control. The green fluorescent protein (GFP) expression unit was linked to the circ-Ccnb1, which contained a separate CMV promoter.

### Routine in vitro and in vivo assays

Cell proliferation and survival were performed as described [36]. In single cell proliferation assay, cells transfected with circ-Ccnb1 and control vector were inoculated in Petri dishes with DMEM containing 10% FBS, which allowed the cells to attach but not spread as tissue culture plates did. The cultures were briefly treated with trypsin/EDTA in the following day to harvest single cell suspension. The cell number was determined to obtain a density of 1 cell per 100 µl followed by immediate distribution into 96-well tissue culture plates, at the amount of 100 µl per well. The wells that contained 1 single cell were used. Cell number was determined daily. Cell migration and colony formation were performed as described [14, 37].

For protein analysis, Western blot was performed as described [38]. Flow cytometry, immunohistochemistry (IHC) and Immunofluorescence microscopy were performed as described [39, 40]. For RNA analysis, Real-time PCR was performed using U6 as an internal control as described [41]. Immunoprecipitation assays included circ-Ccnb1 probe that pulled down circ-Ccnb1 and proteins, and an antibody that pulled down circ-Ccnb1, using the methods described [13]. Northern blot was performed as described [42].

Tumor formation assay was described previously [43]. In the tumor formation assay, 4-week old CD-1 nude mice were randomly divided into 2 groups. Each group had 10 or 11 mice. All mice were injected subcutaneously with MDA-MB-231 cells (5 × 10^6^ cells/mouse), followed by monitoring tumor sizes. The assay was repeated twice. In mouse survival assay, 4-week old CD-1 nude mice were randomly divided into three groups: control vector, injection with circ-Ccnb1 expression plasmid, and injection with circ-Ccnb1-transfected cells. Each group had ten mice. The assay was repeated three times. All mice were injected intraperitoneally with MDA-MB-231 cells (2 × 10^6^ cells/mouse), followed by monitoring mouse survival. In vivo delivery of circ-Ccnb1 plasmids and circ-Ccnb1 siRNAs were performed using the methods developed in the lab [14].

### Proteomic analysis

Proteins binding with circ-Ccnb1 were precipitated by the circ-Ccnb1 probe, followed by Liquid chromatography tandem-mass spectrometry (LC-MS/MS). In brief, the circ-Ccnb1-transfected HTB126 and Beas2B cells were lysed with co-IP buffer containing 20 mM Tris-HCl pH 7.5, 150 mM NaCl, 1 mM EDTA, 0.5% NP-40, and protein inhibitors. After sonication and centrifugation, protein amounts were brought to 10 μg for each group and incubated with 10 μg biotinylated probes against circ-Ccnb1 at room temperature for 2 h. Then, 50 μl Streptavidin C1 magnetic beads (Invitrogen) were pre-washed and added to each binding reaction. The mixtures were further incubated at room temperature for 1 h. The beads were washed with co-IP buffer five times followed by three times with the buffer not containing detergent. The bound proteins were then digested from the beads for LC-MS/MS. The LC-MS/MS results were searched against the human Uniprot database.

### Docking simulations, contact maps and identification of binding site residues

RNA-binding residues in H2AX were predicted by RNABindRPlus (http://ailab1.ist.psu.edu/RNABindRPlus/) [44], RBscore (http://ahsoka.u-strasbg.fr/rbscore/), and Pprint (http://www.imtech.res.in/raghava/pprint/) [45] web tools. The predictions of RNA-binding residues were based on the amino acid environment, biochemical features, and the propensity of various amino acid residues for RNA-binding residues that are calculated from the known 3-D structures of protein–RNA complexes. Residues that were predicted to bind to RNA by at least two of these methods and the side chains that were accessible at the surface were mapped onto H2AX. Based on these predictions and the electrostatic surface potential calculation studies, the H2AX was modeled with the circ-Ccnb1.

To discover the possible interaction of circ-Ccnb1 with H2AX (C-terminal tail), 20,000 models were generated using NPDock server [46], a protein-RNA docking analysis tool. Distance-based and residue-level resolution contact maps of circ-Ccnb1-H2AX (C-terminal tail) docked complex was determined using RNAmap2D [47] and COCOMAPS [48] tools. Contact distances were computed between Cα atoms of protein residues and O5’ atoms of RNA strands. Two residues are in contact when their O5’–Cα distance is less than 10 Å. The distance based approach was used to identify the binding site residues/nucleotides for the protein–RNA complexes using a specific cut-off value. Two atoms (one in RNA and another in protein) are considered to be interacting with each other if the distance between them is < 3.5 Å. Docking analysis of circular RNA-protein interaction has been employed in our previous studies [14, 49].

### Statistical analysis

All experiments were performed in triplicate and numerical data were subject to independent sample *t* test. The levels of significance were set at *p < 0.05 and **p < 0.01. Error bars represent SD (*n* = 4 unless indicated otherwise).

## Electronic supplementary material

circ-CCNB1-p53-Supplementary-March 13, 2018
