# Supplementary figures and images for: Enhanced breast cancer progression by mutant p53 is inhibited by the circular RNA circ-Ccnb1
Source: Cell Death Differ. 2018 May 23;25(12):2195–208. doi: 10.1038/s41418-018-0115-6 (PMC6261950; doi:10.1038/s41418-018-0115-6)

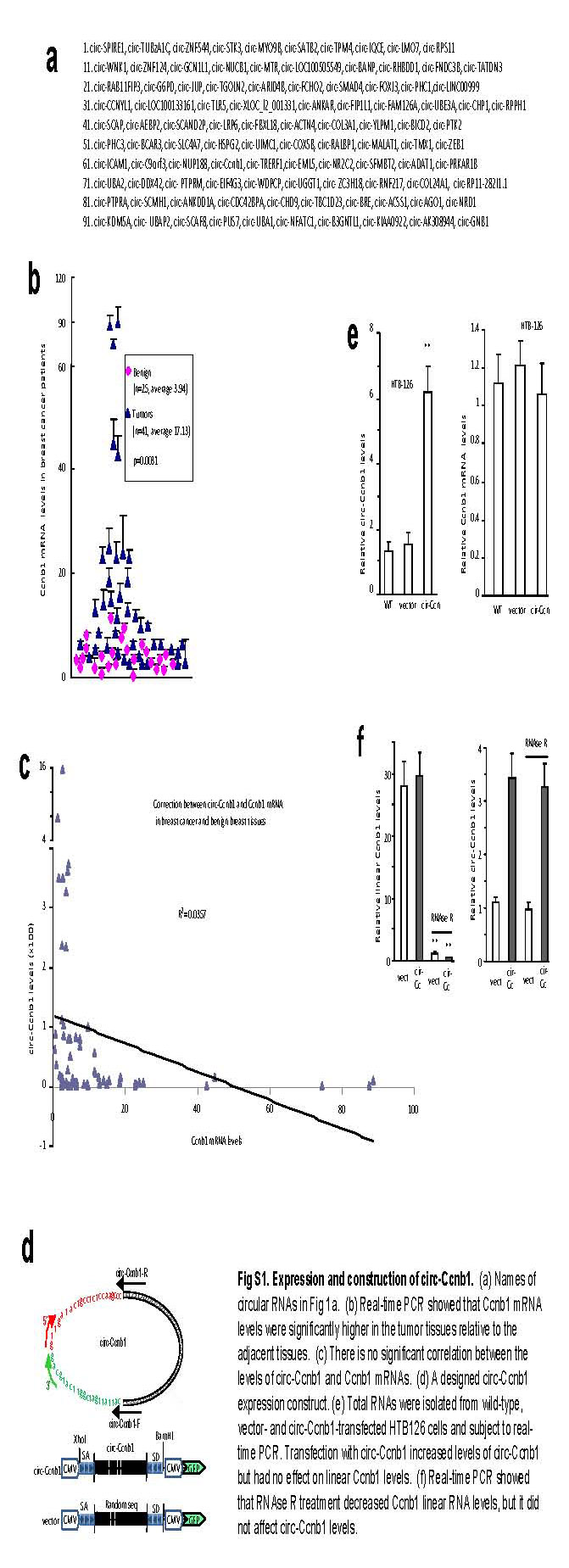


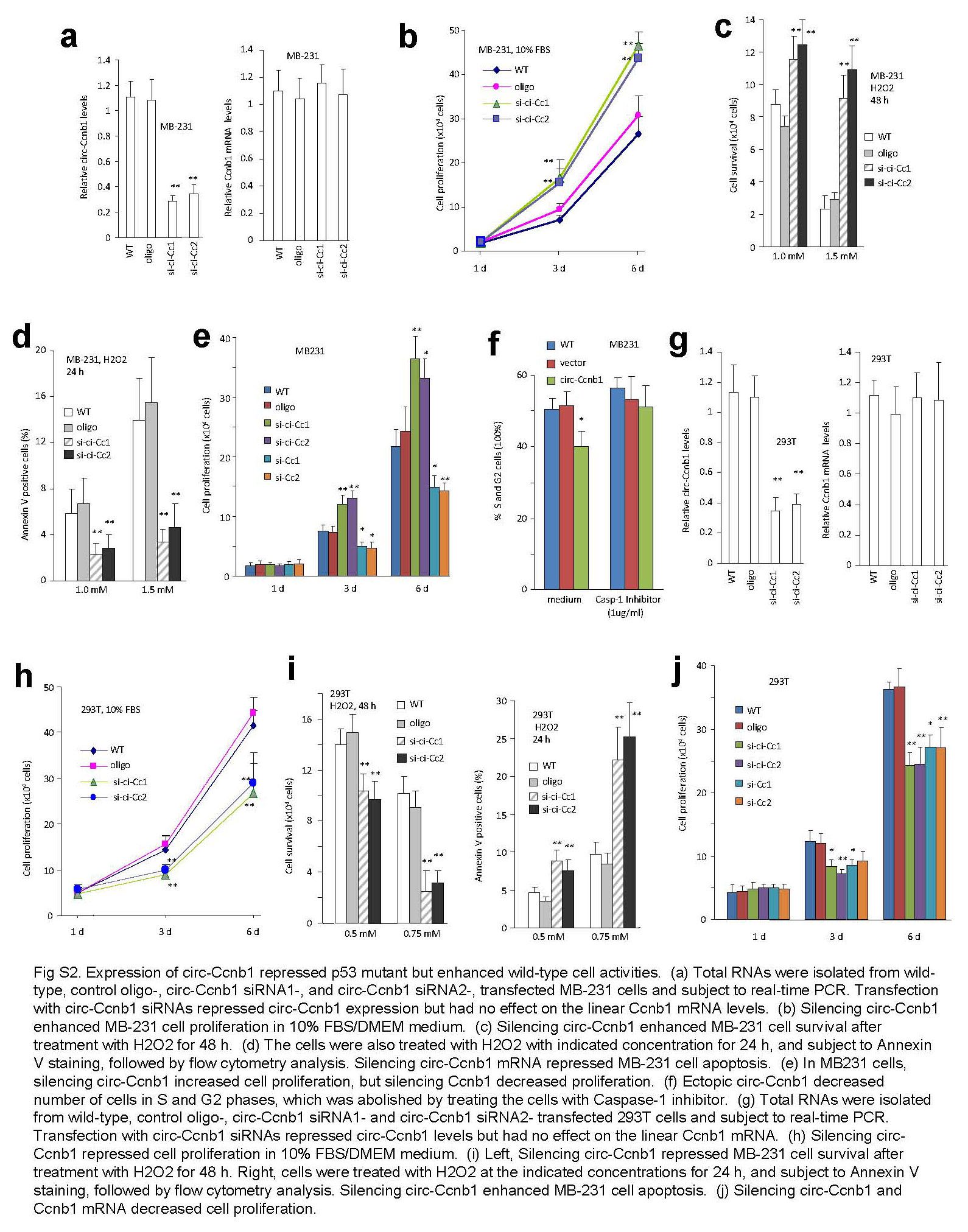


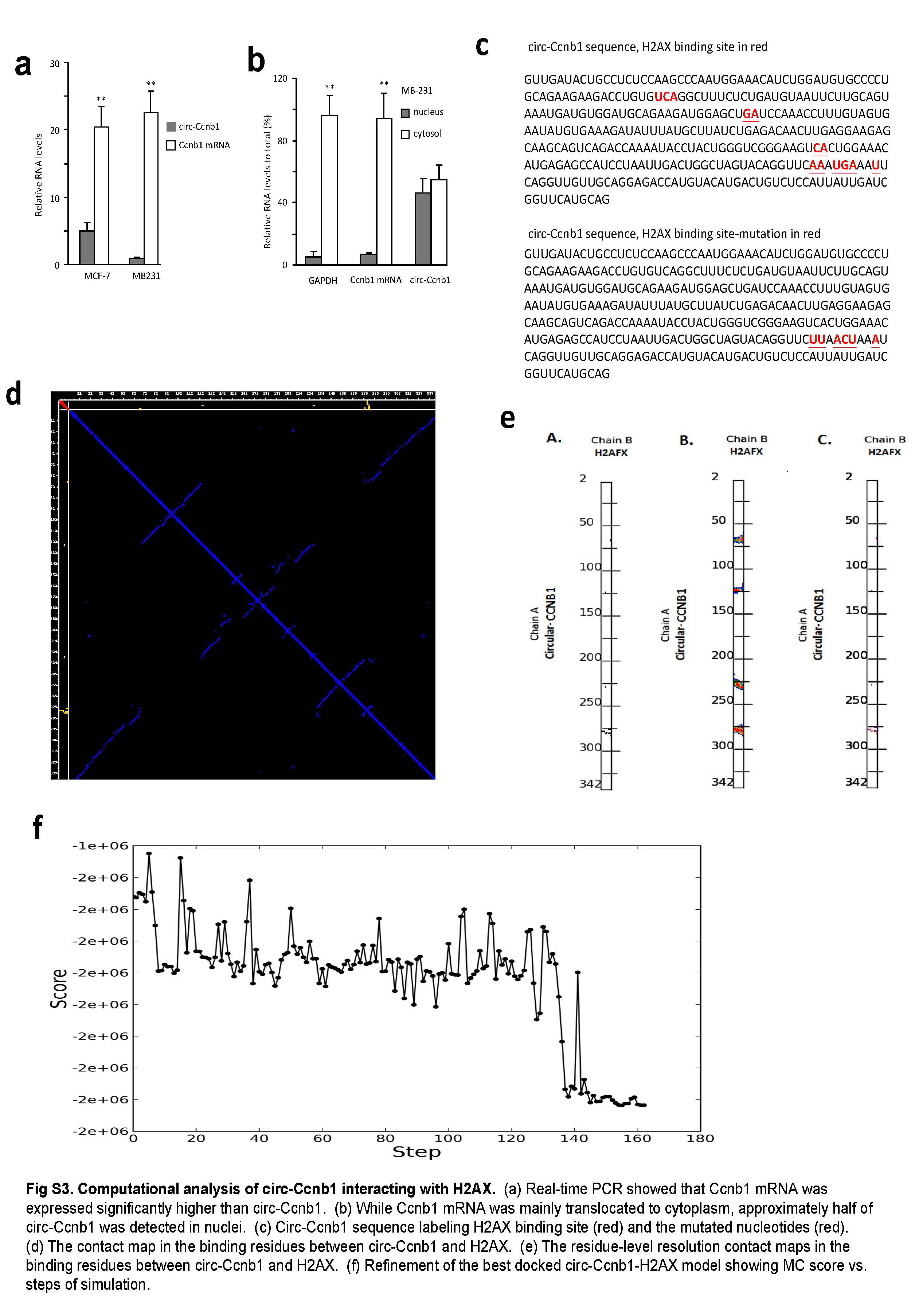


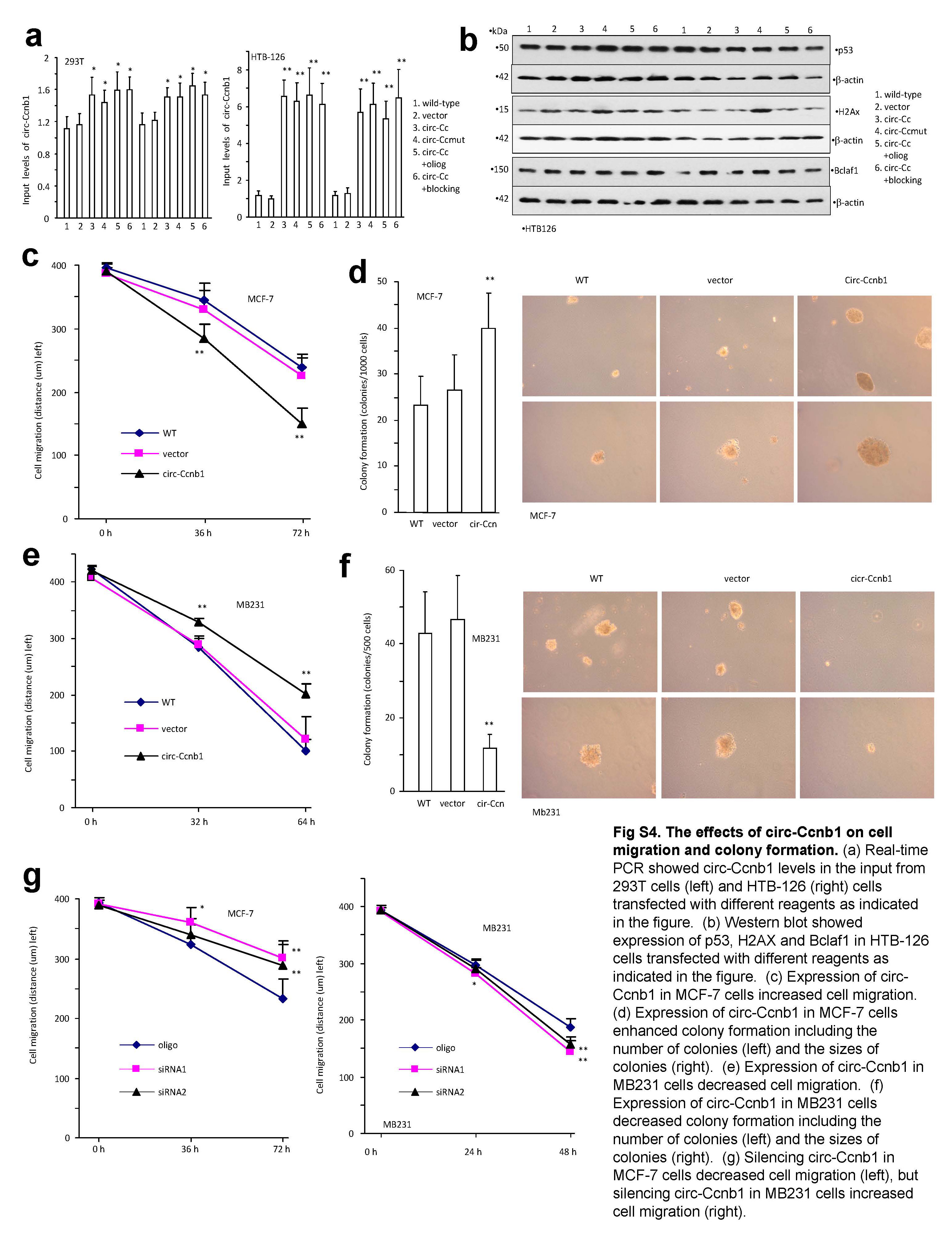


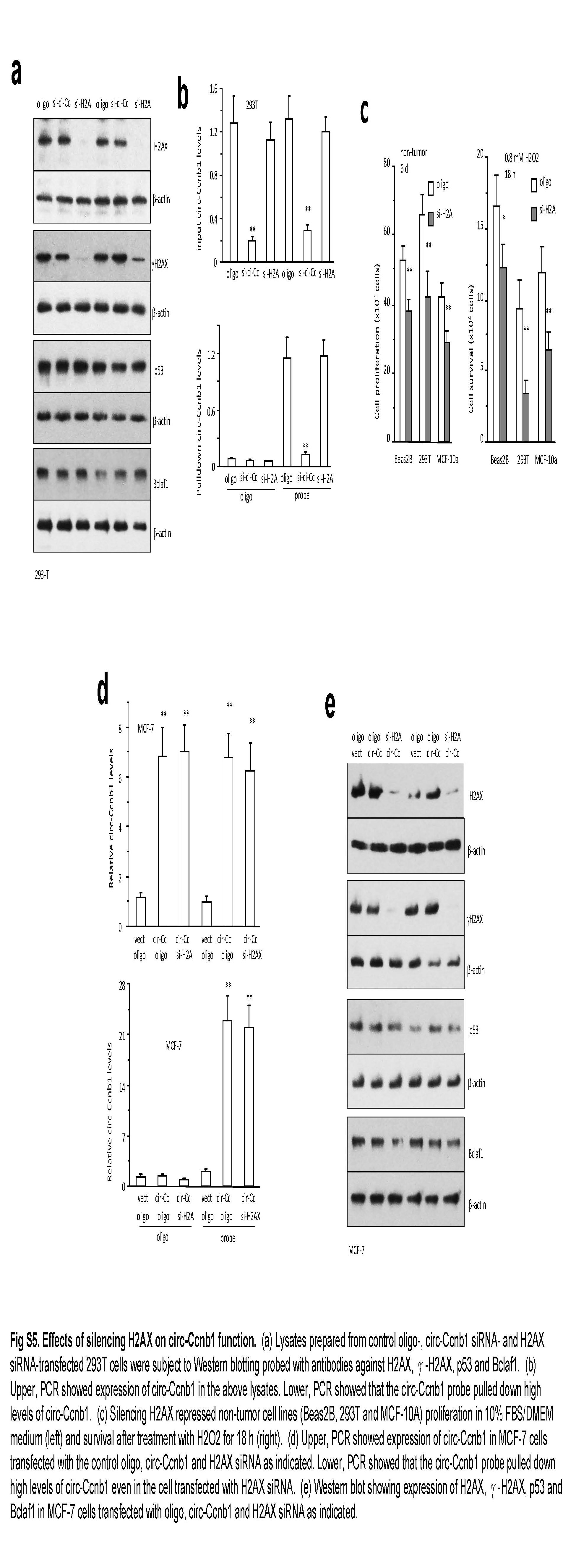


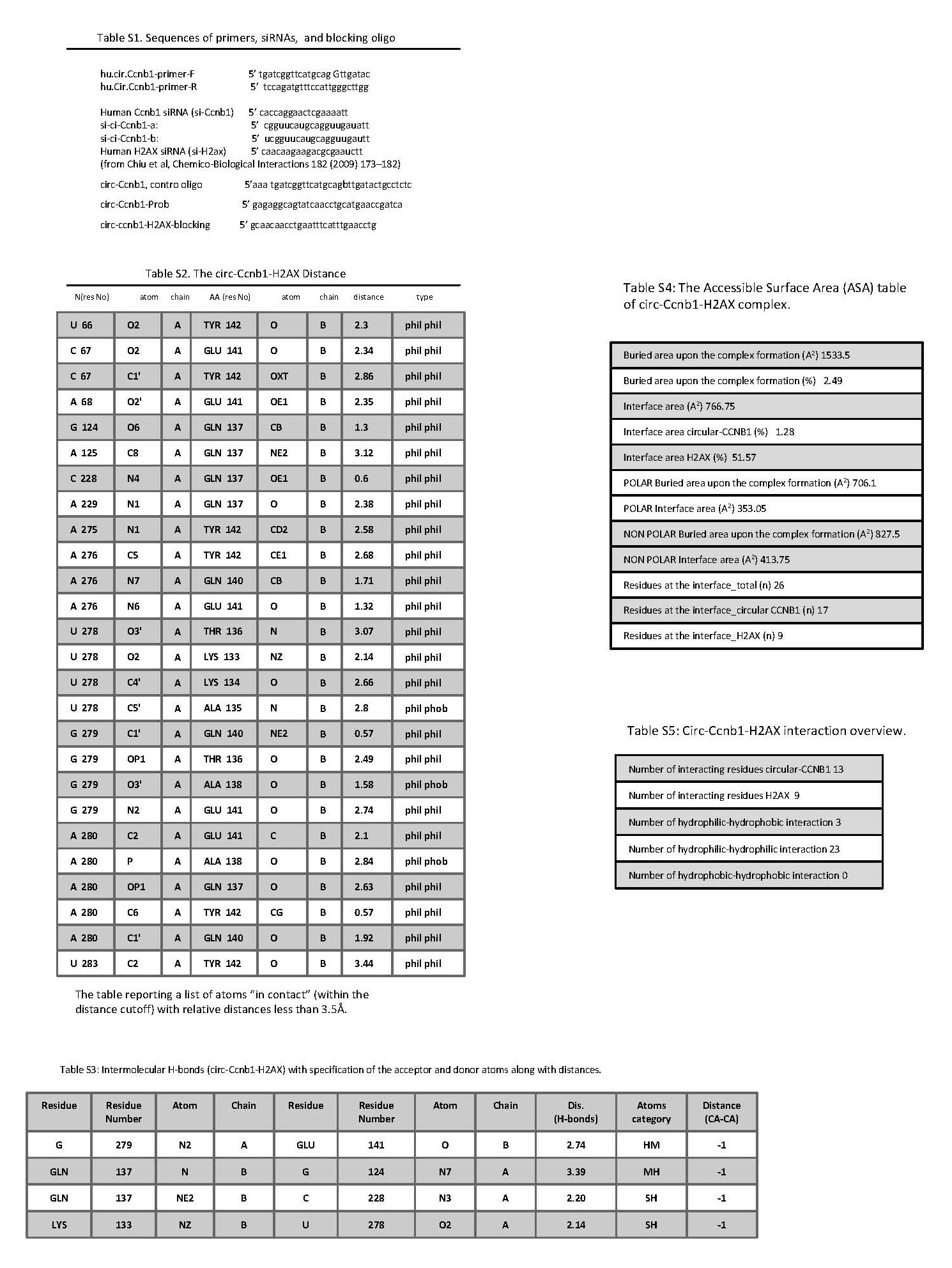

Supplement: Supplementary file 1 — circ-CCNB1-p53-Supplementary-March 13, 2018 [file 41418_2018_115_MOESM1_ESM.doc]
